# Medical Expenditures Associated with Attention-Deficit/Hyperactivity Disorder Among Adults in the United States by Age, 2015–2019

**DOI:** 10.1007/s11606-023-08075-w

**Published:** 2023-02-13

**Authors:** Brian Witrick, Donglan Zhang, Dejun Su, Yan Li, William V. McCall, Brian Hendricks, Lu Shi

**Affiliations:** 1 West Virginia Clinical and Translational Sciences Institute, PO Box 9102, Morgantown, WV 26506 USA; 2 Division of Health Services Research, Department of Foundations of Medicine, New York University Long Island School of Medicine, Mineola, NY USA; 3 University of Nebraska Medical Center, Omaha, NE USA; 4 Icahn School of Medicine at Mount Sinai, New York, NY USA; 5 Medical College of Georgia at Augusta University, Augusta, USA; 6 Department of Epidemiology and Biostatistics, West Virginia University, Morgantown, WV USA; 7 Clemson University, Clemson, SC USA

**Keywords:** attention-deficit/hyperactivity disorder, ADHD, adults, health care costs, medical expenditures

## Abstract

**Background:**

Attention-deficit hyperactivity disorder is a common disorder that affects both children and adults. However, for adults, little is known about ADHD-attributable medical expenditures.

**Objective:**

To estimate the medical expenditures associated with ADHD, stratified by age, in the US adult population.

**Design:**

Using a two-part model, we analyzed data from Medical Expenditure Panel Survey for 2015 to 2019. The first part of the model predicts the probability that individuals incurred any medical costs during the calendar year using a logit model. The second part of the model estimates the medical expenditures for individuals who incurred any medical expenses in the calendar year using a generalized linear model. Covariates included age, sex, race/ethnicity, geographic region, Charlson comorbidity index, insurance, asthma, anxiety, and mood disorders.

**Participants:**

Adults (18 +) who participated in the Medical Expenditure Panel Survey from 2015 to 2019 (*N* = 83,776).

**Main Measures:**

Overall and service specific direct ADHD-attributable medical expenditures.

**Key Results:**

A total of 1206 participants (1.44%) were classified as having ADHD. The estimated incremental costs of ADHD in adults were $2591.06 per person, amounting to $8.29 billion nationally. Significant adjusted incremental costs were prescription medication ($1347.06; 95% CI: $990.69–$1625.93), which accounted for the largest portion of total costs, and office-based visits ($724.86; 95% CI: $177.75–$1528.62). The adjusted incremental costs for outpatient visits, inpatient visits, emergency room visits, and home health visits were not significantly different. Among older adults (31 +), the incremental cost of ADHD was $2623.48, while in young adults (18–30), the incremental cost was $1856.66.

**Conclusions:**

The average medical expenditures for adults with ADHD in the US were substantially higher than those without ADHD and the incremental costs were higher in older adults (31 +) than younger adults (18–30). Future research is needed to understand the increasing trend in ADHD attributable cost.

## Introduction

Attention-deficit hyperactivity disorder (ADHD) is a neuropsychiatric disorder that is normally diagnosed in childhood but may persist into adulthood. According to the Diagnostic and Statistical Manual of Mental Disorders (*DSM-5*).^1^^,^^2^ ADHD is characterized by a repeated set of inattentive or hyperactive-impulsive behaviors that occurs throughout multiple settings and impairs social, academic, or occupational functionality.^3^ An estimated 10–60% of children with ADHD continue to experience symptoms throughout their lives.^4^ ADHD is a common psychiatric disorders experienced by adults,^5^ with a prevalence around 4.4%,^6^ and is one of the most commonly treated disorders in young adults.^7^

The negative social, emotional, and medical outcomes associated with ADHD have been well documented.^8^^,^^9^ Individuals with ADHD have poorer relationships with their family and friends^10–12^ are more likely to engage in illegal activities as they grow older,^13^ and struggle with self-esteem and overall satisfaction in life.^14–16^ In adulthood, individuals with ADHD are more likely to be incarcerated,^17^ divorced,^18^ or unemployed.^19^ Furthermore, ADHD has also been associated with several comorbid conditions, such as depression, anxiety, and substance abuse disorder that increase the disease burden on individuals.^20^

ADHD can pose a significant financial burden on individuals and families, through increased medical costs and productivity loss.^21–26^ Previous studies conducted in the US have found significantly higher medical costs for children/adolescents with ADHD than those without it.^22^ However, few US studies have examined ADHD-related medical expenditures in adults or examined how the cost changes throughout adulthood. Given the increase in the prevalence of ADHD over the years,^27^ updated information is needed about the specific direct medical costs associated with ADHD in adults.

The purpose of this study is to estimate the medical expenditures associated with ADHD, stratified by aged, in the US adult population.

## Methods

### Data Source

Data were obtained from the Medical Expenditure Panel Survey (MEPS) for 2015–2019. MEPS data are a nationally representative sample of the US civilian noninstitutionalized population^28^. Detailed information on the MEPS data collection and sample design is provided by the Agency for Healthcare Research and Quality (AHRQ)^29^. Briefly, for households that participated in the study, 5 rounds of interviews were conducted within 2 years to collect a broad range of information about all members in the household. The survey collects information on self-reported sociodemographic characteristics, health status, health conditions, medical diagnosis, insurance status, use of healthcare services, expenditures for different types of services, and sources of payment. The occurrence of health conditions is determined primarily by prompting respondents of the household component for the causes of medical events and disability episodes for themselves or their family members or asking them about any health conditions that are bothering the person or their family members during the reference period^30^. These conditions are then coded by medical professionals, regardless of whether any medical expenses occurred in that year.

Due to the de-identified data, this study was exempt from institutional review board approval, per the US Department of Health and Human Services guidelines.

### Study Sample

The inclusion criteria for this study was men and women aged 18 years or older. Individuals who reported a diagnosis of ADHD (ICD-9 = 314 or ICD-10 = F90) for the period January 1, 2015, to December 31, 2019, were classified as ADHD.^31^^,^^32^ After excluding individuals with missing values on key variables, the final sample included 83,776 adults.

### Measures

The dependent variables for this study were the direct overall medical expenditures and the service specific medical expenditures. This included hospital stays, outpatient care, emergency room visits, physician office visits, home care visits, and prescription medication. Household members were asked about which medical services each family member used and the corresponding medical costs that occurred. These costs included respondents’ out of pocket payments and payments from private/public health insurance but did not include over-the-counter drugs, as these were not collected by MEPS. Following the guidelines recommend by AHRQ, costs were inflated to the recent estimate of the 2019-dollar value using the Personal Health Care Price Indices.^33^

From the literature, two groups of variables, patient characteristics and medical conditions, were identified as potential predictors of the medical expenditures of ADHD. Patient characteristics included age, gender, race/ethnicity, geographic region, marital status, employment status, education, poverty, and insurance type.^5^^,^^25^ Depending on the year the data was collected, medical conditions were defined using ICD-10-CM codes or the clinical classification codes generated from Clinical Classification Software (CCS).^34^ Medical conditions included an adapted Charlson Comorbidity Index (CCI), asthma (CCS = 128 or ICD-10 = J45), anxiety disorders (anxiety, social phobia, and obsessive–compulsive disorder) (CCS = 651 or ICD-10 = F40/F41), and mood disorders (depression, bipolar) (CCS = 657 or ICD-10 = J31/J32).^4^^,^^5^ The CCI is a commonly used method of categorizing medical comorbidities of patients based on their ICD codes.^35^ The CCI score was calculated using Stata’s Charlson package, which is based on the adaptations of Deyo and Quan to accommodate ICD-9-CM codes and ICD-10-CM codes.^36–38^ For this study, the CCI was grouped into 3 categories: 0, 1, ≥ 2. Asthma was included as a covariate in the model due the high comorbidity found between ADHD and asthma.^4^ Although the exact nature of the association is not fully understood, numerous studies have observed the association in adults and children, and in population and clinical settings.^39^ Anxiety disorders and mood disorders have also been found to have a significant association with ADHD in adults and were thus included in the final model.^5^^,^^40^ Alcohol- and drug-related abuse were not adjusted in the model as previous research found that ADHD generally precedes these behaviors, and that ADHD can contribute to their development.^41,42^

### Data Analytic Procedures

Bivariate analysis was conducted between individuals with ADHD and those without ADHD using chi-square statistics for categorical variables and *t*-statistics for continuous variables. Next, a two-part model was used to estimate the overall and service specific medical expenditures associated with ADHD in US adults. A two-part model was chosen due to the large number of individuals who reported zero medical costs within the calendar year.^43^^,^^44^ The first part of the model predicts the probability that individuals incurred any medical costs during the calendar year using a logit model. The second part of the model estimates the medical expenditures for individuals who incurred any medical expenses in the calendar year using a generalized linear model. The modified park test was used to select the distribution and variance function for the generalized linear model, resulting in gamma or Poisson distribution and a log link function. Covariates included in both parts of the model were age, sex, race, ethnicity, geographic region, insurance type, CCI, asthma, anxiety, and mood disorders. Stratified analysis was conducted by age for younger adults (18-30) and older adults (31 +).

Based on the two regression models, per-person expenditures associated with ADHD were calculated through several steps.^45^ First, assuming everyone had ADHD (A), second assuming everyone did not have ADHD (B), and finally determining the incremental costs of ADHD by subtracting the predicted costs when everyone did not have ADHD from the predicted costs when everyone did have ADHD (A-B). This difference was then multiplied by the average number of individuals with ADHD to estimate the national economic burden of ADHD. The number of individuals with ADHD was estimated directly from MEPS by applying sampling weights, using all possible individuals. Sampling weights were taken into account in all cost estimation analysis, which were 5-year pooled sampling weights averaged by the number of panels (*N* = 5). The bootstrap method with 100 replicates was used to derive standard errors (SEs) and 95% confidence intervals (CIs).

All analyses were conducted using Stata software (version 15.1, StataCorp, College Station, TX) and statistical significance was determined at a *p*-value of 0.05.

## Results

In the study population (Table 1), 1.44% (1206/83,776) were identified as having ADHD. Individuals in the study population were primarily female (51.70%), were employed (63.40%), and had high income (44.52%), and there was no statistically significant differences between the groups for these variables. Overall, the mean unadjusted annual medical expenditures were $6853.91 (SE: 99.21) for all individuals in the study population, with individuals with ADHD ($8589.61 ± 641.94) having significantly higher expenditures than those without ADHD ($6821.72 ± 98.25; *p* = 0.005). When only including patients who had a medical expenditure, the overall unadjusted mean medical expenditures was $8009.25 (SE: 111.43), and the difference between for those with ADHD ($8697.59 ± 649.89) vs those without ADHD ($7994.45 ± 110.54) was insignificant.

**Table 1:** Sample characteristics of study subjects aged 18 + by ADHD status, 2015–2019 Medical Expenditure Panel Survey

	Total (*n* = 83,776)	With ADHD (*n* = 1206)	Without ADHD (*n* = 82,570)	*p* value
Age, mean (SE)	47.58 (0.14)	34.63 (0.49)	47.82 (0.15)	< 0.001
Total expenditures, mean (SE)	6853.91 (99.21)	8589.61 (641.94)	6821.72 (98.25)	0.005
Total expenditures for those with positive expenditures, mean (SE)	81,009.25 (111.43)	8697.59 (649.89)	7994.45 (110.54)	0.27
Gender (%)				0.81
Male	48.30	48.75	49.29	
Female	51.70	51.25	51.71	
Race/ethnicity (%)				< 0.001
Non-Hispanic White	62.95	82.14	62.59	
Non-Hispanic Black	11.78	3.77	11..93	
Hispanic	16.20	7.96	16.35	
Other	9.07	6.13	9.13	
Insurance (%)				< 0.001
Private	69.92	76.57	69.80	
Public	21.93	20.40	21.96	
No insurance	8.14	3.03	8.24	
Marital status (%)				< 0.001
Currently married	51.94	33.34	52.28	
Not currently married	48.06	66.66	47.72	
Geographic region (%)				< 0.001
Northeast	17.66	16.36	17.69	
Midwest	20.87	27.91	20.74	
South	37.72	36.91	37.73	
West	23.76	18.82	23.85	
Education (%)				0.004
No high school degree	12.31	14.53	12.27	
High school or GED	41.73	35.14	41.85	
Other degree	13.62	16.67	13.56	
Bachelor’s degree or higher	31.69	33.13	31.66	
Unknown	0.65	0.49	0.65	
Employment status (%)				0.092
Employed	63.40	66.68	63.34	
Unemployed	36.60	33.32	36.66	
Poverty (%)				0.12
Poor or near poor	14.64	16.17	14.61	
Low income	12.46	10.01	12.51	
Middle income	28.38	26.68	28.41	
High income	44.52	47.15	44.47	
Charlson comorbidity index (%)				0.019
0	83.50	82.75	83.51	
1	11.70	14.25	11.66	
2 +	4.80	3.00	4.84	
Asthma (%)	6.76	11.73	6.67	< 0.001
Anxiety (%)	11.63	34.82	11.20	< 0.001
Mood (%)	10.72	34.28	10.28	< 0.001

Individuals with ADHD were significantly different from those without ADHD regarding patient characteristics and medical conditions. For patient characteristics, individuals with ADHD were more likely to be younger (34.63 vs. 47.82, *p* < 0.001), more likely to be White (82.14% vs. 62.59%, *p* < 0.001), and more likely to have private insurance (76.57% vs. 69.80%, *p* < 0.001). In contrast, individuals without ADHD were more likely to be married (52.28% vs 33.34%, *p* < 0.001) and have the highest educational attainment be a high school degree or equivalent (41.85% vs. 35.14%, *p* = 0.004). Medically, individuals with ADHD were more likely to report asthma, mood disorders, and anxiety.

After controlling for covariates, individuals with ADHD were 12.30 times more likely (95% CI: 6.82–22.20; *p* < 0.001) than individuals without ADHD to have an expenditure of at least $1, and among individuals with positive expenditures, those with ADHD had 28% (exp[0.25]) higher expenditures than individuals without ADHD (β = 0.25; 95% CI: 0.10–0.40; *p* = 0.001) (Table 2). The total incremental costs of ADHD were estimated at $2591.06 (95% CI: $1471.71–$4684.57) (Table 3). The adjusted incremental costs in prescription medication accounted for the largest portion of the total costs, at $1347.06 (95% CI: $990.69–$1625.93). The only other statistically significant adjusted incremental cost was for office-based visits ($724.86; 95% CI $177.75–$1528.62). When estimating costs at the national level, a total of $8.29 billion was attributable to adult ADHD, with $4.31 billion specifically attributable to ADHD prescription costs.

**Table 2:** Results of two-part model of factors associated with total cost of ADHD

	Logit			GLM	
	Estimate	OR	*p* value	Estimate	*p* value
ADHD (reference: non-ADHD)	2.51 (1.92, 3.10)	12.30	< 0.001	0.25 (0.10, 0.40)	0.001
Age	0.03 (0.07, 0.03)	1.03	< 0.001	0.02 (0.01, 0.02)	< 0.001
Female (reference: male)	0.70 (0.65, 0.76)	2.01	< 0.001	0.10 (0.06, 0.14)	< 0.001
Race/ethnicity (reference: non-Hispanic White)					
Non-Hispanic Black	− 0.51 (− 0.60. − 0.41)	0.60	< 0.001	− 0.02 (− 0.10, 0.06)	0.631
Hispanic	− 0.57 (− 0.65, − 0.49)	0.57	< 0.001	− 0.14 (− 0.21, − 0.07)	< 0.001
Other	− 0.52 (− 0.63, − 0.40)	0.59	< 0.001	− 0.21 (− 0.30, − 0.11)	< 0.001
Insurance (reference: private)					
Public	− 0.08 (− 0.18, 0.01)	0.92	0.093	− 0.05 (− 0.10, 0.01)	0.094
No insurance	− 1.38 (− 1.47, − 0.23)	025	< 0.001	− 0.71 (− 0.83, − 0.59)	< 0.001
Not currently married (reference: currently married)	− 0.12 (− 0.19, − 0.06)	0.89	< 0.001	− 0.04 (− 0.09, 0.01)	0.143
Geographic region (reference: Northeast					
Midwest	0.23 (0.11, 0.35)	1.26	< 0.001	− 0.00 (− 0.09, 0.09)	0.983
South	− 0.05 (− 0.14, 0.05)	0.95	0.339	− 0.09 (− 0.16, − 0.01)	0.020
West	0.02 (− 0.09, 0.13)	1.02	0.760	− 0.07 (− 0.15, 0.01)	0.096
Education (reference: Bachelor’s Degree or Higher)					
No high school degree	− 0.46 (− 0.56, − 0.37)	0.6	< 0.001	− 0.17 (− 0.25, − 0.08)	< 0.001
High school or GED	− 0.50 (− 0.58, − 0.42)	0.61	< 0.001	− 0.05 (− 0.12, 0.01)	0.111
Other degree	− 0.30 (− 0.40, − 0.20)	0.74	< 0.001	0.00 (− 0.09, 0.09)	0.968
Unknown	− 1.35 (− 1.63, − 1.07)	0.26	< 0001	0.24 (− 0.08, 0.56)	0.139
Employed (reference: unemployed)	− 0.19 (− 0.26, − 0.13)	0.83	< 0.001	− 0.36 (− 0.42, − 0.31)	< 0.001
Poverty (reference: poor or near poor)					
Low income	0.06 (− 0.04, 0.16)	1.06	0.241	− 0.09 (− 0.16, − 0.01)	0.021
Middle income	0.14 (0.04, 0.23)	1.15	0.005	− 0.07 (− 0.14, − 0.01)	0.035
High income	0.36 (0.26, 0.46)	1.43	< 0.001	− 0.01 (− 0.08, 0.07)	0.902
Charlson comorbidity index (reference: 0)					
1	2.35 (2.09, 2.60)	10.49	< 0.001	0.66 (0.59, 0.74)	< 0.001
2 +	4.33 (3.45, 5.20)	75.94	< 0.001	1.17 (1.08, 1.25)	< 0.001
Asthma	0.21 (− 0.10, 0.52)	1.23	0.186	− 0.14 (− 0.21, − 0.06)	0.001
Anxiety	1.53 (1.34, 1.73)	4.62	< 0.001	0.33 (0.26, 0.39)	< 0.001
Mood	1.45 (1.26, 1.64)	4.26	< 0.001	0.43 (0.36, 0.49)	< 0.001

**Table 3:** Estimated per-capita and annual medical expenditure by costs category associated with ADHD

	Predicted Costs with ADHD (A)	Predicated costs Without ADHD (B)	Expenditure Difference (A-B)	National Annual Expenditure Associated with adult ADHD (Billions)
Total direct costs	9616.08 (8340.09, 10,876.02)	7025.02 (6894.72, 7181.07)	2591.06 (1471.71, 4684.57)*	8.29 (4.71, 14.98)
Inpatient visits	1494.19 (981.40, 234.07)	1685.97 (1588.23, 1754.02)	− 191.78 (− 707.11, 601.36)	− 0.61 (− 2.26, 1.94)
Prescription medication	3009.45 (2616.97, 3259.54)	1662.39 (1602.13, 1718.73)	1347.06 (990.69, 1625.93)*	4.31 (3.17, 5.20)
Outpatient visits	1105.17 (580.70, 1534.12)	638.58 (606.53, 672.53)	466.59 (− 29.70, 912.64)	1.49 (− 0.10, 2.92)
Emergency room visits	251.78 (169.10, 305.98)	256.94 (242.91, 268.53)	− 5.16 (− 79.56, 46.60)	− 0.02 (− 0.25, 0.15)
Office − based visits	2392.36 (1833.92, 3192.67)	1667.50 (1624.34, 1704.32)	724.86 (177.75, 1528.62)*	2.32 (0.57, 4.89)
Home health visits	446.59 (217.22, 723.55)	335.87 (303.25, 373.27)	110.71 (− 112.33, 373.99)	0.35 (− 0.36, 1.20)

Estimated 3.20 million adults with ADHD

^*^Statistically significant at *p* < 0.05

The adjusted odds of having a medical expenditure of at least $1 were 9.21 times higher (95% CI: 4.66–17.99; *p* < 0.001) in individuals with ADHD than individuals without ADHD in adults 18–30 and 28.50 times higher (95% CI: 8.50–91.84; *p* < 0.001) in adults 31 + (Table 4). Among individuals with positive medical expenditures, adults 18–30 with ADHD had 39% higher expenditures (β = 0.33; 95% CI: 0.03–0.63; *p* = 0.029) than adults without ADHD and adults 31 + with ADHD had 25% higher expenditures (β = 0.22; 95% CI: 0.04–0.41; *p* = 0.018). The total incremental cost was higher in adults 31 + than among younger adults 18–30, $2623.48 (95% CI: $997.94–$5012.65) and $1856.66 (95% CI: $864.01–3,455.02) respectively (Table 5). Incremental expenditures associated with prescription medication accounted for the largest portion of the total costs in both age groups and was higher in older adults aged 31 + ($1339.74; 95% CI: $707.62–$1796.89) than younger adults aged 18–30 ($722.10; 95% CI: $493.06–$1029.96). Conversely, younger adults with ADHD had higher adjusted incremental expenditures for office base visits ($627.28; 95% CI: $322.09–$1507.26) than older adults ($449.53; 95% CI: $130.034–$945.71). The adjusted incremental expenditures for inpatient visits, outpatient visits, emergency room visits, and home health visits were not statistically different in either age group.

**Table 4:** Results of two-part model of factors associated with total cost of ADHD by age group

	Adults 18–30					Adults 31 +				
	Logit			GLM		Logit			GLM	
	Estimate	OR	*p* value	Estimate	*p* value	Estimate	OR	*p* value	Estimate	*p* value
ADHD (reference: non-ADHD)	2.22 (1.54, 2.89)	9.21	< 0.001	0.33 (0.03, 0.63)	0.029	3.35 (2.14, 4.57)	28.50	< 0.001	0.22 (0.04, 0.41)	0.018
Age	− 0.03 (− 0.05, − 0.01)	0.97	< 0.001	0.02 (− 0.00, 0.04)	0.118	0.04 (0.03, 0.04)	1.04	< 0.001	0.01 (0.00, 0.01)	< 0.001
Female (reference: male)	0.87 (0.77, 0.98)	2.39	< 0.001	0.30 (0.17, 0.43)	< 0.001	0.60 (0.53, 0.66)	1.82	< 0.001	0.03 (− 0.01, 0.08)	0.146
Race/ethnicity (reference: non-Hispanic White)										
Non-Hispanic Black	− 0.64 (− 0.80, − 0.49)	0.53	< 0.001	0.26 (− 0.04, 0.56)	0.090	− 0.38 (− 0.49, − 0.27)	0.68	< 0.001	− 0.09 (− 0.15, − 0.02)	0.009
Hispanic	− 0.73 (− 0.86, − 0.60)	0.48	< 0.001	0.03 (− 0.18, 0.23)	0.785	− 0.39 (− 0.48, − 0.30)	0.68	< 0.001	− 0.20 (− 0.27, − 0.12)	< 0.001
Other	− 0.51 (− 0.70, − 0.32)	0.60	< 0.001	− 0.06 (− 0.27, 0.16)	0.604	− 0.50 (− 0.62, − 0.37)	0.61	< 0.001	− 0.24 (− 0.33, − 0.15)	< 0.001
Insurance (reference: private)										
Public	− 0.12 (− 0.26, 0.02)	0.89	0.084	− 0.05 (− 0.25, 0.16)	0.655	− 0.07 (− 0.19, 0.05)	0.93	0.275	− 0.07 (− 0.12, − 0.03)	0.003
No insurance	− 1.20 (− 1.34, − 1.05)	0.30 −	< 0.001	− 0.62 (− 0.87, − 0.38)	< 0.001	− 1.37 (− 1.48, − 1.26)	0.25	< 0.001	− 0.79 (− 092, − 0.66)	< 0.001
Not currently married (reference: currently married)	− 0.40 (− 0.55, − 0.25)	0.67	< 0.001	− 0.29 (− 0.44, − 0.15)	< 0.001	− 0.15 (− 0.22, − 0.07)	0.86	< 0.001	0.08 (0.02, 0.13)	0.004
Geographic region (reference: Northeast										
Midwest	0.24 (0.04, 0.43)	1.27	0.016	0.13 (− 0.06, 0.31)	0.187	0.21 (0.08, 0.33)	1.23	0.002	− 0.04 (− 0.13, 0.06)	0.469
South	− 0.09 (− 0.24, 0.07)	0.91	0.261	− 0.05 (− 0.24, 0.14)	0.622	− 0.05 (− 0.16, 0.07)	0.95	0.403	− 0.10 (− 0.17, − 0.03)	0.004
West	0.01 (− 0.15, 0.17)	1.01	0.909	0.03 (− 0.18, 0.23)	0.812	0.02 (− 0.12, 0.15)	1.02	0.807	− 0.09 (− 0.17, − 0.01)	0.023
Education (reference: Bachelor’s degree or higher)										
No high school degree	− 0.62 (− 0.84, − 0.40)	0.54	< 0.001	− 0.09 (− 0.36, 0.18)	0.512	− 0.66 (− 079, − 0.54)	0.52	< 0.001	− 0.09 (− 0.18, − 0.00)	0.047
High school or GED	− 0.68 (− 0.86, − 0.50)	0.51	< 0.001	− 0.00 (− 0.17, 0.16)	0.959	− 0.52 (− 0.61, − 0.42)	0.59	< 0.001	− 0.03 (− 0.10, 0.04)	0.365
Other degree	− 0.52 (− 0.73, − 0.32)	0.59	< 0.001	− 0.05 (− 0.24, 0.15)	0.641	− 0.27 (− 0.40, − 0.14)	0.76	< 0.001	0.03 (− 0.07, 0.13)	0.530
Unknown	− 1.10 (− 1.73, − 0.47)	−	< 0.001	0.84 (0.04, 1.65)	0.040	− 1.50 (− 1.81, − 1.19)	0.22	< 0.001	0.13 (− 0.21, 0.47)	0.438
Employed (reference: unemployed)	− 0.05 (− 0.16, 0.06)	−	0.376	− 0.34 (− 0.48, − 0.20)	< 0.001	− 0.13 (− 0.23, − 0.04)	0.88	0.004	− 0.44 (− 0.50, − 0.38)	< 0.001
Poverty (reference: poor or near poor)										
Low income	− 0.01 (− 0.19, 0.17)	0.99	0.906	0.07 (− 0.14, 0.29)	0.509	0.06 (− 0.05, 0.18)	1.06	0.283	− 0.10 (− 0.18, − 0.02)	0.019
Middle income	0.05 (− 0.10, 0.21)	1.05	0.502	0.01 (− 0.17, 0.19)	0.909	0.14 (0.02, 0.26)	1.15	0.022	− 0.06 (− 0.13, 0.02)	0.143
High income	0.14 (− 0.03, 0.31)	1.15	0.111	0.06 (− 0.17, 0.30)	0.589	0.12 (0.29, 0.55)	1.13	< 0.001	0.04 (− 0.05, 0.12)	0.381
Charlson comorbidity index (reference: 0)										
1	1.86 (1.29, 2.44)	6.42	< 0.001	0.65 (0.31, 0.99)	< 0.001	2.49 (2.19, 2.78)	12.06	< 0.001	0.65 (0.58, 0.73)	< 0.001
2 +	3.09 (1.55, 4.62)	21.98	< 0.001	1.37 (0.76, 1.98)	< 0.001	4.41 (3.41, 5.42)	82.27	< 0.001	1.16 (1.08, 1.24)	< 0.001
Asthma	0.48 (− 0.11, 1.06)	1.62	0.112	− 0.15 (− 0.49, 0.20)	0.399	0.17 (− 0.24, 0.58)	1.19	0.427	− 0.13 (− 0.21, − 0.06)	0.001
Anxiety	1.46 (1.11, 1.82)	4.31	< 0.001	0.37 (0.20, 0.55)	< 0.001	1.60 (1.34, 1.85)	4.95	< 0.001	0.29 (0.22, 0.36)	< 0.001
Mood	1.74 (1.39, 2.09)	5.70	< 0.001	0.54 (0.37, 0.70)	< 0.001	1.36 (1.14, 1.59)	3.90	< 0.001	0.37 (0.31, 0.44)	< 0.001

**Table 5:** Estimated incremental per-capita medical expenditures associated with ADHD by age group

	Adults 18–30	Adults 31 +
Total direct costs	1856.66 (864.01, 3455.02)*	2623.48 (997.94, 5012.65)*
Inpatient visits	− 81.04 (− 412.89, 323.68)	50.05 (− 195.53, 395.05)
Prescription medication	722.10 (493.06, 1029.96)*	1339.74 (707.62, 1796.89)*
Outpatient visits	127.46 (− 112.74, 421.12)	383.64 (− 6.01, 1244.48)
Emergency Room visits	− 65.93 (− 131.26, 0.78)	37.58 (− 60.88, 129.53)
Office-based visits	627.28 (322.09, 1507.26)*	449.53 (130.034, 945.71)*
Home health visits	82.36 (− 107.74, 2091.24)	50.05 (− 195.53, 395.05)

Estimated 1.56 million adults 18–30 with ADHD and 1.66 million adults 31 + with ADHD

^*^Statistically significant at *p* < 0.05

## Discussion

The aim of this study was to estimate the medical expenditures associated with ADHD, stratified by age, in the US adult population. Overall, the estimated incremental cost of ADHD in adults was $2591.06 per person, amounting to $8.29 billion nationally. In older adults (31 +), the incremental cost of ADHD was $2623.48, while in younger adults (18-30) the incremental cost was $1856.66. The largest proportion of medical expenditures across all age groups was associated with prescription medication.

Our study demonstrates that individuals with ADHD incurred higher medical expenditures compared to individuals without ADHD. The estimated incremental costs of ADHD per person found in this study are consistent with previous studies conducted in the US, highlighting the continued impact of ADHD on medical expenditures.^4,5^^,^^46^ In 2011, Hodgkins et al. analyzed data from two large healthcare claims and productivity databases and found adults with ADHD incurred an excess of $2100.76 (adjusted to 2019-dollar) more in medical expense.^5^ More recently, Shah and Onukwugha analyzed MEPS data from 2017 to 2018 and found that working adults with ADHD incurred $4328 more than working adults without ADHD.^46^ Although their findings of the total costs of ADHD are higher than ours, this is likely due to the differences in inclusion criteria for their study sample, which was restricted to adults who were employed in the study year. Employment status has a significant impact of healthcare utilization and costs, with unemployed individuals less likely to visit physicians for preventative services and less likely to fill prescriptions.^47^^,^^48^ This impact may be even greater in individuals diagnosed with ADHD, as ADHD medication can be expensive and often requires frequent visits to the physician for proper management.^49^^,^^50^ By including all adults, regardless of insurance or employment, our findings allow for a more complete understanding of the financial impact of ADHD in adults.

On a national level, the estimated $8.29 billion for total medical costs is consistent with findings from previous studies of $1.32–$39.79 billion (adjusted to 2019-dollar value).^25^ These results indicate that the medical cost of ADHD has a substantial economic impact in the US and policy initiatives might be needed to alleviate this economic burden. This is especially troubling since ADHD also results in additional costs to society, such productivity and income loss, education, and justice system.^25^^,^^51^ Furthermore, although the ADHD prevalence found in our study, 1.44%, is similar to the prevalence found in a recent study using Kaiser Permanente data^27^, 1.12%, it is less than the 4.4% that is commonly cited by other studies.^25^^,^^51^ Therefore, our estimated medical expenditures associated with ADHD at the national level are a relatively conservative assessment.

In this study, stratification into two age groups provided additional insights into ADHD medical expenditures. We found that ADHD medical expenditures were higher in older adults (31 +) than younger adults (18-30), which is consistent with a recent study conducted in Germany.^52^ This extra burden of medical expenditure among older adults with ADHD might reflect the lifetime accumulative burden of illness from ADHD, a topic which deserves more academic and clinical attention (e.g., from the perspective of chronic disease management, healthy aging among older adults with ADHD, healthcare cost-saving among older adults with ADHD). More research is needed to understand why the incremental cost is so much higher in older adults than in younger adults and how to address this increased cost.

In our study, prescription medication was the largest component of the total medical expenditures for ADHD for all adults, and within each age group. These findings are consistent with previous studies of adult and children/adolescent populations.^23^^,^^46^ Although there are several treatment options for individuals with ADHD, pharmacological treatments, i.e. medications, are the most commonly utilized.^53^ Consequently, this leads to higher medical expenditures due to prescription medication in individuals with ADHD. However, other potential treatment options, such as mindfulness-based cognitive therapy, need to be explored more fully.^54^ Unfortunately, the majority of cost-effectiveness research on the treatment of ADHD has been focused on children.^53^ Our findings indicate that more cost-effectiveness research for ADHD treatment is needed in adults to provide affordable, high-quality care for everyone with ADHD.

As compared with past studies, one of the main strengths of our study is that it used a large, nationally representative sample to estimate medical expenditures, including individuals with and without insurance and nonworking adults. This allows for the fact that individuals with ADHD could have difficulty with employment, which affects their likelihood of having insurance and thus did not enter the data sources provided by payers.^55^ Another strength of MEPS data is that it includes the total payments respondents reported which were paid. Finally, this study used a two-part model to estimate the medical expenditures. The two-part model has a long history in health economics and health services research due to its ability to model continuous, non-negative outcome measures.^56^ The model is able to handle data that has a skewed conditional distribution, a large number of zero values, and allows for investigation of the effects of demographic and clinical comorbidities on both the logit model and GLM model separately.^56^^,^^57^

This study has several limitations. First, the MEPS data is based on self-reported responses and only includes ADHD if an individual is currently being managed for the disorder or it is identified as a current problem. This might explain why our estimates of ADHD rate (1.44%) are lower than some worldwide estimates (1.0–5.4%).^58^ Second, current DSM-5 criteria for ADHD have been criticized for having low clinical thresholds, which results in a misclassification of individuals who do not experience the symptoms of ADHD being classified with the diagnosis.^59^^,^^60^ In our study, this would lead to an underestimation of the medical expenditures associated with ADHD since not all individuals in the ADHD cohort would truly have the diagnosis. Third, our national cost estimates were likely to be underestimated because MEPS did not include people who were incarcerated. This is important because the ADHD rates have been reported as high as 25.5% in prisoners.^40^ Fourth, as MEPS data is cross-sectional, the results can only be interpreted as medical expenditures associated with ADHD and cannot account for costs associated with ADHD over a lifetime. Finally, our study did not include indirect cost associated with ADHD, such as productivity and income loss, education, and justice system.

## Conclusion

ADHD is a common psychiatric disorder that impacts a large number of adults in the US. We found that the average medical expenditures for adults with ADHD in the US were substantially higher than those without ADHD and the incremental costs were higher in older adults (31 +) than in younger adults (18-30).

## Data Availability

The datasets analyzed for this study are publicly available and are provided by the Agency for Healthcare Research and Quality (https://www.meps.ahrq.gov/mepsweb/).
